# Inhibitory Effect of Veterinary Antibiotics on Denitrification in Groundwater: A Microcosm Approach

**DOI:** 10.1155/2014/879831

**Published:** 2014-03-16

**Authors:** Mahtab Ahmad, Meththika Vithanage, Kangjoo Kim, Ju-Sik Cho, Young Han Lee, Young Kyoo Joo, Sang Soo Lee, Yong Sik Ok

**Affiliations:** ^1^Department of Biological Environment, Kangwon National University, Chuncheon 200-701, Republic of Korea; ^2^University Institute of Biochemistry and Biotechnology, PMAS Arid Agriculture University, Rawalpindi 46000, Pakistan; ^3^Chemical and Environmental Systems Modeling Research Group, Institute of Fundamental Studies, 20000 Kandy, Sri Lanka; ^4^Department of Environmental Engineering, Kunsan National University, Gunsan 573-701, Republic of Korea; ^5^Department of Bio-Environmental Sciences, Sunchon National University, Suncheon 540-950, Republic of Korea; ^6^Division of Plant Environmental Research, Gyeongsangnam-do Agricultural Research and Extension Service, Jinju 660-360, Republic of Korea; ^7^Division of Biological Science and Technology, Yonsei University, Wonju 220-710, Republic of Korea

## Abstract

Veterinary antibiotics in groundwater may affect natural microbial denitrification process. A microcosm study was conducted to evaluate the influence of sulfamethazine and chlortetracycline at different concentrations (0, 0.01, 0.1, and 1.0 mg/L) on nitrate reduction in groundwater under denitrifying condition. Decrease in nitrate removal and nitrite production was observed with the antibiotics. Maximum inhibition of nitrate removal was observed after seven days of incubation with 0.01 mg/L sulfamethazine (17.0%) and 1.0 mg/L chlortetracycline (15.4%). The nitrite production was inhibited with 1.0 mg/L sulfamethazine to 82.0% and chlortetracycline to 31.1%. The initial/final nitrate concentrations indicated that 0.01 mg/L sulfamethazine and 1.0 mg/L chlortetracycline were most effective in inhibiting activity of denitrifying bacteria in groundwater. After 12 days of incubation, the sulfamethazine biodegradation was observed whereas chlortetracycline was persistent. Sulfamethazine and chlortetracycline in groundwater could inhibit the growth and capability of naturally occurring denitrifying bacteria, thereby threatening nitrate pollution in groundwater.

## 1. Introduction

An antibiotic is a chemical substance produced by selected microorganisms that are capable of inhibiting the growth of other microorganisms [1]. Significant advances in medicines and public health have escalated the development of antibiotics to combat infectious diseases in humans and animals [2]. The worldwide consumption of antibiotics is estimated about 100,000 to 200,000 t/y [3]. Generally, the medicated antibiotics are designed to be quickly excreted from the animal body, resulting in 60 to 90% release into the surrounding environment [3, 4]. Therefore, sewage sludge and animal manure can be primary sources of antibiotics' release or contamination of soil and groundwater [5].

The use of veterinary antibiotics in livestock industries has increased tremendously due to their benefits towards growth promotion and disease prevention in food animals [6]. Korea ranked one of the biggest consumers of veterinary antibiotics in Asia with 1,533 t/y [7]. Since a large proportion of these veterinary antibiotics remains in unmetabolized status in the environments via animal urine and feces, the manure is considered to be the most viable vehicle of veterinary antibiotics into the environment [8]. Recently, Ok et al. [9] monitored seven veterinary antibiotics, including chlortetracycline, oxytetracycline, tetracycline, sulfamethazine, sulfamethoxazole, sulfathiazole, and tylosin, near a manure composting facility in Gangwon Province, Korea. The authors reported that surface water, soil, and sediment of this area were contaminated with these veterinary antibiotics compared to an area outside of the composting site. Extensive use of antibiotics-laden manure from the livestock industry on cropland also provides a route of entry for veterinary antibiotics into the groundwater system [10, 11]. However, little information is available on the occurrence and fate of veterinary antibiotics in groundwater [12, 13]. In fact, antibiotics may have the potential to be more persistent than organic pollutants due to their constant release into the aquatic ecosystem [14]. Furthermore, there is much concern over infections from newly developed strains of antibiotic resistant microorganisms posing a potential threat to humans and animals. The antibiotic resistant genes in these newly transformed species can be considered emerging contaminants for which mitigation strategies are needed to prevent their widespread distribution [15].

Nitrate contamination in groundwater systems is a worldwide problem associated with the intensive applications of nitrogen fertilizers and manure to agricultural fields [16]. Most of the rural areas in Korea rely on local groundwater for drinking purposes because of a lack of centralized water supply system [17]. However, nitrate contamination level of groundwater in some areas is exceeding the national drinking water restriction of 10 mg/L nitrate nitrogen (NO_3_-N) [17]. It is apprehensive of a number of health risks such as methemoglobinemia, hypertension, infant mortality, goiter, stomach cancer, thyroid disorder, cytogenetic defects, and birth defects [18].

Denitrification is a bacterially facilitated process to dissimilate nitrate to nitrogen gas via a series of intermediate steps [19]. In groundwater systems, natural denitrification may occur in the presence of sufficient organic carbon by facultative heterotrophic bacteria. However, it is very likely that the release of antibiotics from manure into groundwater may have a deleterious effect on naturally occurring denitrifying bacteria, consequently extending the residence of nitrate contamination in groundwater.

To date, the effects of antibiotics in groundwater systems on nitrate denitrification have not been well known although it is of utmost importance. The objective of this study is to determine the effects of two veterinary antibiotics of sulfamethazine and chlortetracycline in groundwater on the nitrate denitrification by bacteria under anaerobic conditions in microcosm experiment.

## 2. Methods

### 2.1. Synthetic Groundwater and Bacteria Inoculum

Synthetic groundwater was prepared using calcium chloride (CaCl_2_), sodium bicarbonate (NaHCO_3_), magnesium sulfate (MgSO_4_ *·*7H_2_O), and sulfuric acid (H_2_SO_4_) in deionized water [20].  Table 1 shows the chemical composition of synthetic groundwater. The synthetic groundwater was spiked with 50 mg/L NO_3_-N to obtain a concentration level five times higher than the standard limit (10 mg/L NO_3_-N) for drinking water in Korea [19]. The synthetic groundwater was autoclaved at 121°C for 20 min to sanitize before use. All chemicals were employed as a high purity analytical reagent (AR) grade. Deionized water was produced from a water purification system (Arium Pro UV/DI Water Purification System; Sartorius Stedium Biotech, Göttingen, Germany).

Heterotrophic microbial cultures were grown in the laboratory under anaerobic conditions using natural groundwater. These cultures were used to prepare the inoculum in synthetic groundwater. Ethanol, acted as a source of organic carbon for microbial growth, and the inoculum were enriched with heterotrophic microbes at 25°C for seven days.

### 2.2. Microcosm Experiment

Microcosms were prepared in sterile glass serum bottles to investigate the inhibitory effects of two antibiotics (sulfamethazine and chlortetracycline) on the nitrate reduction by microbes. These two antibiotics were selected as representatives of sulfonamide and tetracycline groups of antibiotics, which are widely used in Korea [9]. A triplicate set of serum bottles was prepared containing 200 mL synthetic groundwater and 5 mL inoculum. Nitrogen gas was purged for 10 min to remove dissolved oxygen and the bottles were sealed with rubber septa. Sulfamethazine and chlortetracycline were injected at the concentrations of 0.01, 0.1, and 1.0 mg/L into the respective serum bottles. Acetylene gas was injected into each serum bottle to inhibit the reduction of N_2_O to N_2_ [21]. Finally, all tests including the control (without antibiotics) were incubated using a mechanical shaking incubator rotating at 70 rpm in the dark at 25°C.

### 2.3. Determination of Nitrate and Nitrite Concentration

An aliquot was taken from each serum bottle using a sterile syringe every day and then filtered through a 0.45 *μ*m membrane filter for measuring nitrate and nitrite concentrations using an ion chromatography (Metrohm Compact IC-861, Switzerland). The eluent was prepared as a mixture of 3.2 mM Na_2_CO_3_ and 1.0 mM NaHCO_3_ and was degassed before use. A 20.0 mM H_2_SO_4_ was used as a regeneration solution. The instrument was calibrated on a daily basis using appropriate standard solutions. For antibiotics analysis, an aliquot was periodically sampled from each serum bottle. The sampled solution was also filtered through a 0.45 *μ*m membrane filter and then transferred to amber colored vials. A high performance liquid chromatographer (HPLC; SCL-10A, Shimadzu, Japan) equipped with an autosampler (SIL-10AD; Shimadzu, Japan) and a UV-VIS detector (SPD-10A; Shimadzu, Japan) was used. A reverse-phase Sunfire C18 column (4.6 mm × 250 mm; Waters, USA) was employed in a column oven (CTO-10AS; Shimadzu, Japan). The mobile phase A was a mixture of 99.9% deionized water and 0.1% formic acid, while mobile phase B comprised 99.9% acetonitrile and 0.1% formic acid. The eluent was pumped as 70% mobile phase A and 30% mobile phase B in a binary gradient mode at the rate of 0.32 mL/min. A 10 *μ*L aliquot of the sampled solution was injected into the column at 25°C and the absorbance was measured at 254 nm.

### 2.4. Statistical Analysis

Statistical analysis was done using the statistical analysis system (SAS, ver.9.3, Cary, NC, USA). Mean values of three replicates were subjected to one-way analysis of variance (ANOVA) and Tukey's honestly significant difference (HSD) test at a 0.05 significance level.

## 3. Results and Discussion

### 3.1. Denitrification

A heterotrophic medium culture was used to ensure the denitrification process because facultative heterotrophic bacteria consume nitrate in the absence of dissolved oxygen and precede denitrification process in the following steps: NO_3_ → NO_2_ → NO → N_2_O → N_2_.  Table 2 shows the partial denitrification process with/without antibiotics at various rates. Regardless of antibiotics effects, the nitrate concentration rapidly decreased within seven days of incubation and the decreasing rate was reduced until 19 days. Contrarily, the nitrite was rapidly produced and then steadily reduced within the same time span of incubation. These results are in accordance with the theoretical denitrification that the nitrite concentration is increasing at the expense of nitrate under anaerobic conditions [22]. Other studies have also reported similar findings [18, 23].

### 3.2. Effects of Antibiotics on Nitrate Reduction

**Figure 1:**
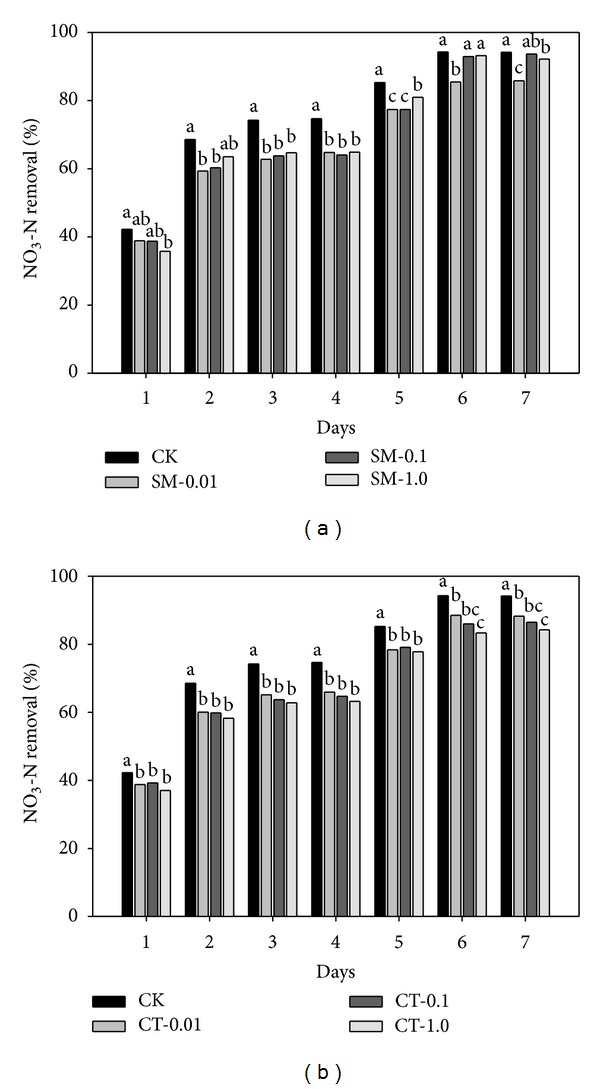
Comparison of nitrate (NO_3_-N) removal over time in groundwater under denitrifying conditions in the absence (CK) or presence of (a) sulfamethazine (SM-0.01, -0.1, and -1.0 mg/L) and (b) chlortetracycline (CT-0.01, -0.1, and -1.0 mg/L). Same letters above each bar indicate no difference between different treatments at a 0.05 significance level of the Tukey's studentized range test.

**Figure 2:**
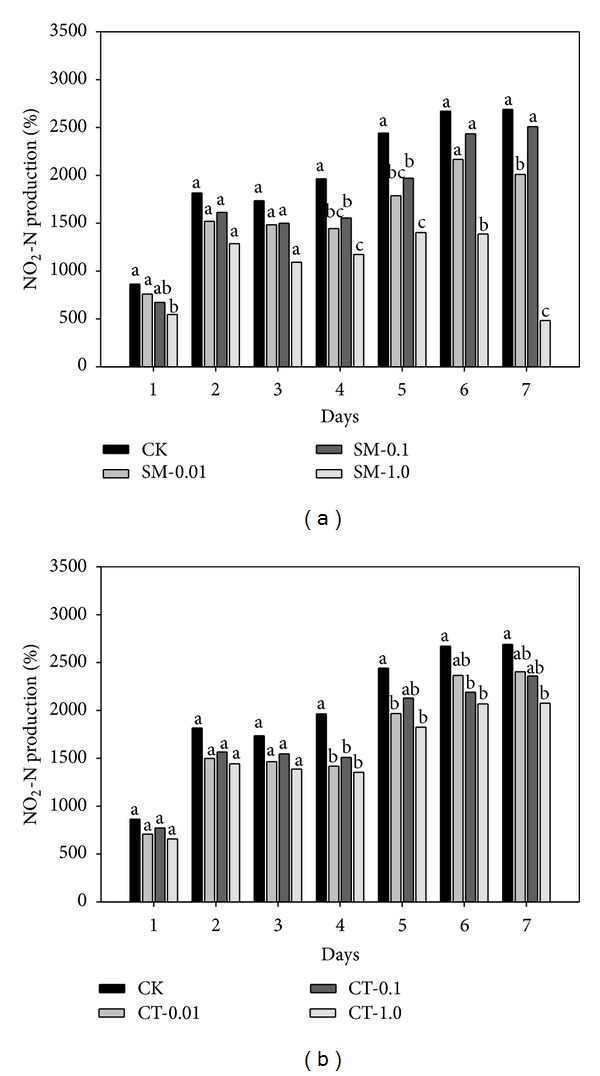
Comparison of nitrite (NO_2_-N) production over time in groundwater under denitrifying conditions in the absence (CK) or presence of (a) sulfamethazine (SM-0.01, -0.1, and -1.0 mg/L) and (b) chlortetracycline (CT-0.01, -0.1, and -1.0 mg/L). Same letters above each bar indicate no difference between different treatments at a 0.05 significance level of the Tukey's studentized range test.

Antibiotics can affect microbial populations and their biochemical metabolism [24]. The microbial activity may either be inhibited or enhanced due to the toxicity or stress of antibiotics [3, 25]. In this study, sulfamethazine and chlortetracycline inhibited the nitrate reduction by microbes in groundwater.  Figure 1 shows the nitrate removal with/without antibiotics during seven days of incubation. The reason for showing partial data only for seven days of incubation is that the maximum reduction in nitrate was observed at this period. During the first seven days, a significant decrease in nitrate removal from groundwater was observed with both antibiotics. A maximum inhibition of 16.99% was observed in the microcosm exposed to 0.01 mg/L sulfamethazine compared to the control whereas 1.0 mg/L chlortetracycline inhibited the nitrate reduction by up to 15.40%. Likewise, the product of nitrite was reduced by up to 82.03% with 1.0 mg/L sulfamethazine and up to 31.08% with 1.0 mg/L chlortetracycline (Figure 2). The higher concentration of chlortetracycline was more effective in decreasing the removal of nitrate or the product of nitrite than that of sulfamethazine. These findings are consistent with a study of Underwood et al. [21] who reported a gradual decrease in the nitrate removal and the nitrite product with increasing dosages of sulfamethoxazole. In this study, after 12 days of incubation, the nitrate reduction was slightly increased in the microcosms exposing the relatively high concentrations of sulfamethazine (0.1 and 1.0 mg/L) compared to the control. Correspondingly, the nitrite product in the same microcosm was rapidly decreased compared to the control. This could possibly be attributed to enhanced microbial activity under stressful condition by antibiotics [3].

The initial nitrate removal rate after one day was 21.88 mg/L/d in the control, which was gradually decreased to 1.72 mg/L/d after 30 days of incubation. The addition of antibiotics led to a decrease of the nitrate removal rate compared to the control. After four days of incubation, the nitrate removal rates exposed to 1.0 mg/L sulfamethazine and chlortetracycline were 8.39 and 8.19 mg/L/d, respectively, compared to 9.67 mg/L/d in the control, resulting in 13.24 and 15.31% reductions of the nitrate removal rate, respectively. Costanzo et al. [25] reported the significant depressions of denitrification rate in groundwater exposed to 1.0 mg/L erythromycin, clarithromycin, and amoxicillin, compared to the control.

**Figure 3:**
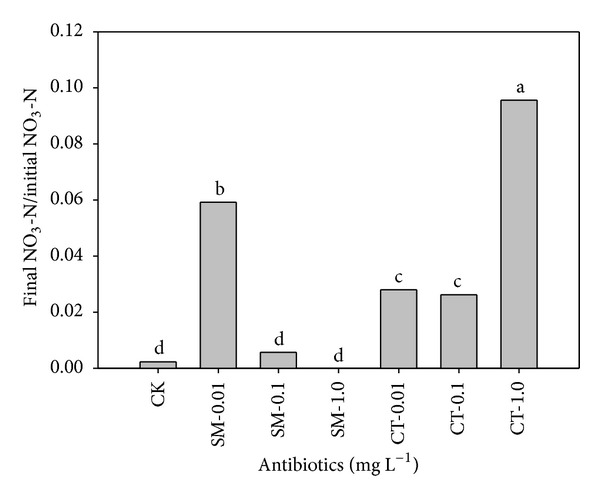
Comparison of final/initial nitrate (NO_3_-N) concentrations for sulfamethazine (SM-0.01, -0.1, and -1.0 mg/L) and chlortetracycline (CT-0.01, -0.1, and -1.0 mg/L) after 19 days of incubation. Same letters above each bar indicate no difference between different treatments at a 0.05 significance level of the Tukey's studentized range test.

To reduce the variability in initial nitrate concentrations among samples, the data were normalized by dividing the final nitrate concentration (after 19 days of incubation) by the initial nitrate concentration.  Figure 3 shows the ratio of the final and initial nitrate concentrations at different addition rates of sulfamethazine and chlortetracycline. The 0.01 mg/L sulfamethazine and 1.0 mg/L chlortetracycline showed the most significant effect of inhibiting the activity of denitrifying bacteria in groundwater, as indicated by the high final/initial nitrate ratios of 0.059 and 0.096, respectively, versus a ratio of 0.002 in the control. Relatively greater inhibition of microorganisms on the nitrate reduction activity at low concentration (0.01 mg/L) of sulfamethazine than chlortetracycline could be due to its greater toxicity. Liu et al. [26] reported that sulfonamides are more toxic than tetracyclines to plants growth and microbial activities. Contrarily, at a high concentration of 1.0 mg/L, chlortetracycline toxicity inhibiting the microbial activity was to be greatly extended, whereas sulfamethazine at same concentration exerted a stress thereby enhancing the microbial denitrifying activity.

The inhibitory effects of sulfamethazine and chlortetracycline on nitrate reduction and nitrite production by heterotrophic bacteria in groundwater suggested that the antibiotics have deleterious effects on denitrifying bacteria. Gram-negative bacteria are most likely to be affected by antibiotics. Particularly,* nitrosomonas* and* pseudomonas* are the most commonly isolated denitrifying bacteria in natural groundwater [19, 25].* Pseudomonas proteolytica* has been reported to be the dominant species in groundwater under denitrifying conditions, whose activity was reduced by increasing dosages of sulfamethoxazole [21]. Another implication is that horizontal gene transfer processes by conjugation, transformation, or transduction can lead to the development of antibiotic resistance species. The pathways of antibiotic resistance microorganisms and the antibiotic resistance genes are very similar to antibiotics [15]. Additionally, even after the cells carrying antibiotic resistance genes are killed, their DNA remains persistent in the environment and can be shared between other microorganisms by horizontal gene transfer [15]. Underwood et al. [21] found an increased ratio of* napA* to* napG* genes of nitrate reductase in groundwater exposed to sulfamethoxazole. It showed the changes in genes sequences that could result in the development of antibiotic resistance microorganisms.

### 3.3. Antibiotics Degradation

**Figure 4:**
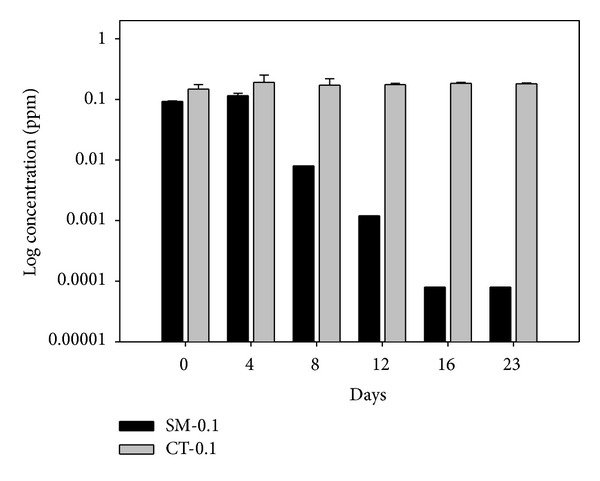
Degradation of sulfamethazine (SM-0.1 mg/L) and chlortetracycline (CT-0.1 mg/L) over time in groundwater under denitrifying conditions.

Concentrations of sulfamethazine and chlortetracycline in microcosms were periodically analyzed to determine the degradation by microbial activity (Figure 4). Sulfamethazine at 0.1 mg/L was significantly degraded after 12 days of incubation. This could be related to the enhanced microbial activity under stressful condition of a relatively high concentration of sulfamethazine, showing the greater nitrate removal and nitrite production (Table 2). Under the stressful condition of antibiotics, the high activity of denitrifying bacteria may result in the degradation of sulfamethazine that can transfer to a new carbon source [8]. However, chlortetracycline did not show any degradation during 23 days of incubation. The possible explanation for the stability of chlortetracycline relies on the formation of Ca- or Mg-ionophores from organic phase complexation [27]. This could further presage to long-term deleterious effects of chlortetracycline on the nitrate reduction by denitrifying bacteria in groundwater.

The present investigation was carried out on synthetic groundwater spiked with nitrates and two different antibiotics in microcosms. For the future implications, a comprehensive study on real groundwater contaminated with both nitrates and antibiotics in the microcosm and continuous column experiments would be worthwhile. Additionally, the changes of the microbial community due to the antibiotics' impacts would further explore the factors involved in the inhibition of denitrification.

## 4. Conclusions

The study was carried out to demonstrate the potential impacts of two selected veterinary antibiotics on natural denitrification process in groundwater. Sulfamethazine and chlortetracycline significantly inhibited the nitrate reduction and nitrite production by denitrifying bacteria under anaerobic conditions in groundwater. At a low concentration of 0.01 mg/L, sulfamethazine more effectively decreased nitrate reduction. However, chlortetracycline showed a greater inhibition of nitrate reduction at a high concentration of 1.0 mg/L. Relatively high concentrations of sulfamethazine (i.e., 0.1 and 1.0 mg/L in this study) seemed to produce stressful conditions to microbes that facilitated their activity, resulting in a greater reduction of nitrates in groundwater. This stressful condition may have also caused biological degradation of sulfamethazine by bacteria whereas chlortetracycline was persistent. The findings of this study indicated the fate of antibiotics on microbial process in groundwater and it demonstrated the potential threats to humans and animals. However, it is worth mentioning that the observations in this study are relative to a controlled microcosm experiment using synthetic groundwater and need to be validated in field conditions.

## Figures and Tables

**Table 1:** Chemical composition of synthetic groundwater.

pH	6.78
Calcium chloride (CaCl_2_) (mg/L)	133.2
Sodium bicarbonate (NaHCO_3_) (mg/L)	208
Magnesium sulfate (MgSO_4_·7H_2_O) (mg/L)	59
Sulfuric acid (H_2_SO_4_) (mg/L)	85
Sodium nitrate (NaNO_3_) (mg/L)	303.6
Ethanol (C_2_H_6_O) (mg/L)	70

**Table 2:** Nitrate (NO_3_-N) and nitrite (NO_2_-N) concentrations over time in groundwater under denitrifying conditions in the absence (CK) or presence of sulfamethazine (SM-0.01, -0.1 and -1.0 mg/L) and chlortetracycline (CT-0.01, -0.1 and -1.0 mg/L).

Days	NO_3_-N (mg/L)							NO_2_-N (mg/L)						
	CK	SM-0.01	SM-0.1	SM-1.0	CT-0.01	CT-0.1	CT-1.0	CK	SM-0.01	SM-0.1	SM-1.0	CT-0.01	CT-0.1	CT-1.0
0	51.78 ± 0.35	51.06 ± 0.85	51.21 ± 0.02	51.21 ± 0.55	50.83 ± 0.81	50.99 ± 0.66	51.10 ± 0.38	0.37 ± 0.11	0.27 ± 0.12	0.33 ± 0.07	0.16 ± 0.04	0.30 ± 0.08	0.33 ± 0.11	0.35 ± 0.08
1	29.90 ± 1.00	31.65 ± 0.65	31.73 ± 1.04	33.27 ± 0.58	31.71 ± 0.50	31.46 ± 0.16	32.61 ± 0.37	3.61 ± 0.50	3.22 ± 0.13	2.90 ± 0.11	2.42 ± 0.29	3.03 ± 0.42	3.27 ± 0.04	2.84 ± 0.22
2	16.29 ± 1.48	21.07 ± 1.86	20.57 ± 0.98	18.89 ± 0.74	20.67 ± 0.66	20.81 ± 0.31	21.61 ± 0.42	7.19 ± 1.26	6.08 ± 0.23	6.43 ± 0.75	5.20 ± 0.63	5.99 ± 0.75	6.25 ± 0.09	5.79 ± 0.42
3	13.33 ± 0.71	19.29 ± 1.85	18.77 ± 1.50	18.29 ± 0.78	18.04 ± 0.74	18.78 ± 0.62	19.26 ± 0.30	6.88 ± 1.54	5.93 ± 0.27	6.00 ± 1.10	4.47 ± 1.00	5.87 ± 0.76	6.17 ± 0.06	5.58 ± 0.51
4	13.12 ± 0.77	18.24 ± 0.58	18.63 ± 1.38	18.21 ± 0.69	17.62 ± 0.79	18.27 ± 1.06	19.03 ± 0.37	7.74 ± 0.64	5.79 ± 0.27	6.21 ± 0.64	4.78 ± 0.33	5.69 ± 0.77	6.04 ± 0.10	5.45 ± 0.52
5	7.62 ± 0.55	11.71 ± 0.4	11.72 ± 0.27	9.86 ± 0.21	11.20 ± 0.71	10.83 ± 0.79	11.49 ± 0.61	9.53 ± 0.47	7.08 ± 0.74	7.76 ± 0.83	5.64 ± 0.09	7.75 ± 0.63	8.36 ± 0.52	7.22 ± 0.68
6	2.97 ± 0.20	7.54 ± 0.42	3.68 ± 0.35	3.54 ± 0.26	5.95 ± 0.64	7.25 ± 1.22	8.62 ± 0.83	10.39 ± 0.87	8.50 ± 0.24	9.50 ± 1.01	5.57 ± 0.58	9.25 ± 0.61	8.58 ± 0.59	8.13 ± 0.34
7	3.01 ± 0.21	7.36 ± 0.56	3.29 ± 0.15	4.05 ± 0.38	6.08 ± 0.66	7.01 ± 0.49	8.16 ± 0.53	10.46 ± 0.90	7.91 ± 0.62	9.78 ± 0.58	2.19 ± 0.37	9.39 ± 0.45	9.22 ± 0.48	8.16 ± 0.43
8	2.93 ± 0.33	7.28 ± 0.48	3.25 ± 0.16	ND*	5.98 ± 0.63	6.99 ± 0.58	8.28 ± 0.39	10.37 ± 0.87	7.75 ± 0.59	9.53 ± 0.74	ND	9.32 ± 0.45	9.19 ± 0.48	8.20 ± 0.26
9	2.94 ± 0.33	7.14 ± 0.44	3.24 ± 0.16	ND	5.94 ± 0.62	6.57 ± 0.31	8.09 ± 0.53	10.35 ± 0.86	7.69 ± 0.48	9.56 ± 0.56	ND	9.38 ± 0.29	9.24 ± 0.62	8.11 ± 0.29
10	0.97 ± 0.21	4.05 ± 0.45	0.40 ± 0.07	ND	3.79 ± 0.50	3.82 ± 0.69	5.78 ± 0.59	8.80 ± 0.44	7.34 ± 0.53	8.93 ± 0.25	ND	9.17 ± 0.55	9.49 ± 0.53	8.43 ± 0.49
11	0.59 ± 0.11	4.32 ± 0.52	0.41 ± 0.06	ND	3.58 ± 0.61	3.63 ± 0.41	5.61 ± 0.57	8.71 ± 0.46	7.09 ± 0.59	8.58 ± 0.35	ND	9.09 ± 0.34	9.19 ± 0.39	8.28 ± 0.32
12	0.16 ± 0.04	2.87 ± 0.28	0.04 ± 0.01	ND	1.39 ± 0.24	1.27 ± 0.17	4.84 ± 0.56	6.80 ± 0.75	6.86 ± 0.88	5.78 ± 0.25	ND	8.41 ± 0.44	9.21 ± 0.75	8.07 ± 0.35
13	0.05 ± 0.01	3.07 ± 0.37	0.05 ± 0.01	ND	1.39 ± 0.30	1.45 ± 0.26	4.96 ± 0.68	6.36 ± 0.74	6.66 ± 0.71	4.94 ± 0.45	ND	8.49 ± 0.53	8.96 ± 0.35	8.41 ± 0.34
14	0.12 ± 0.02	3.06 ± 0.38	0.06 ± 0.01	ND	1.38 ± 0.27	1.12 ± 0.18	4.96 ± 0.63	5.79 ± 0.73	6.61 ± 0.73	2.57 ± 0.27	ND	8.52 ± 0.42	9.14 ± 0.78	8.17 ± 0.50
15	0.07 ± 0.02	3.07 ± 0.42	0.11 ± 0.02	ND	1.45 ± 0.20	0.96 ± 0.37	4.93 ± 0.55	5.80 ± 0.67	6.41 ± 0.65	2.16 ± 0.30	ND	8.33 ± 0.60	9.17 ± 0.73	7.37 ± 0.81
16	0.09 ± 0.02	3.07 ± 0.42	0.16 ± 0.02	ND	1.48 ± 0.19	1.51 ± 0.49	4.93 ± 0.59	5.79 ± 0.70	6.44 ± 0.71	2.11 ± 0.30	ND	8.37 ± 0.60	9.19 ± 0.80	8.32 ± 0.30
17	0.09 ± 0.01	3.02 ± 0.30	0.18 ± 0.06	ND	1.50 ± 0.21	1.55 ± 0.34	4.87 ± 0.68	5.84 ± 0.65	6.43 ± 0.70	2.10 ± 0.30	ND	8.28 ± 0.44	8.95 ± 0.64	7.90 ± 0.72
18	0.10 ± 0.02	3.10 ± 0.39	0.22 ± 0.05	ND	1.38 ± 0.23	1.20 ± 0.23	4.86 ± 0.68	5.79 ± 0.69	6.44 ± 0.75	2.76 ± 0.34	ND	8.13 ± 0.60	8.76 ± 0.53	8.06 ± 0.46
19	0.12 ± 0.02	3.03 ± 0.37	0.29 ± 0.06	ND	1.42 ± 0.09	1.34 ± 0.04	4.89 ± 0.62	5.76 ± 0.66	6.34 ± 0.76	1.99 ± 0.24	ND	8.05 ± 0.56	8.92 ± 0.89	8.30 ± 0.50

*Not detected.
